# Comparison of sedation strategies for critically ill patients: a protocol for a systematic review incorporating network meta-analyses

**DOI:** 10.1186/s13643-016-0338-x

**Published:** 2016-09-20

**Authors:** Brian Hutton, Lisa D. Burry, Salmaan Kanji, Sangeeta Mehta, Melanie Guenette, Claudio M. Martin, Dean A. Fergusson, Neill K. Adhikari, Ingrid Egerod, David Williamson, Sharon Straus, David Moher, E. Wesley Ely, Louise Rose

**Affiliations:** 1 Clinical Epidemiology Program, Ottawa Hospital Research Institute, The Ottawa Hospital, General Campus, 501 Smyth Rd, Ottawa, ON K1H8L6 Canada; 2 School of Epidemiology, Public Health and Preventive Medicine, University of Ottawa, Ottawa, ON Canada; 3 Leslie Dan Faculty of Pharmacy, University of Toronto, Toronto, ON Canada; 4 Department of Pharmacy, Mount Sinai Hospital, 600 University Avenue, Toronto, ON M5G1X5 Canada; 5 Department of Pharmacy, The Ottawa Hospital, Ottawa, ON Canada; 6 Department of Medicine, Interdepartmental Division of Critical Care, University of Toronto, Toronto, ON Canada; 7 Department of Medicine, Division of Critical Care, Mount Sinai Hospital, Toronto, ON Canada; 8 Department of Medicine, Division of Critical Care, Schulich School of Medicine and Dentistry, Western University, London, ON Canada; 9 Lawson Health Research Institute, London Health Sciences Centre, London, ON Canada; 10 Department of Medicine, University of Ottawa, Ottawa, ON Canada; 11 Centre for Practice-Changing Research, Ottawa Hospital Research Institute, Ottawa, ON Canada; 12 Department of Critical Care Medicine, Sunnybrook Health Sciences Centre, Toronto, ON Canada; 13 Evaluative Clinical Sciences, Trauma, Emergency and Critical Care Research Program, Sunnybrook Research Institute, Toronto, ON Canada; 14 University of Copenhagen, Rigshospitalet, Neurointensive Intensive Care, Copenhagen O, Denmark; 15 Faculté de Pharmacie, Université de Montréal, Montreal, QC Canada; 16 Département de Pharmacie, Hôpital du Sacré-Coeur, Montreal, QC Canada; 17 Knowledge Translation Program, Li Ka Shing Knowledge Institute, Saint Michael’s Hospital, Toronto, ON Canada; 18 Department of Medicine, Vanderbilt University Medical Center, Health Services Research Center, Nashville, TN USA; 19 Lawrence S. Bloomberg Faculty of Nursing, University of Toronto, Toronto, ON Canada; 20 Institute for Clinical Evaluative Sciences, Toronto, ON Canada; 21 Provincial Centre of Weaning Excellence, Toronto East General Hospital, Toronto, ON Canada

**Keywords:** Sedation protocol, Daily sedation interruption, Mechanical ventilation, Intensive care, Systematic review, Network meta-analysis

## Abstract

**Background:**

Sedatives and analgesics are administered to provide sedation and manage agitation and pain in most critically ill mechanically ventilated patients. Various sedation administration strategies including protocolized sedation and daily sedation interruption are used to mitigate drug pharmacokinetic limitations and minimize oversedation, thereby shortening the duration of mechanical ventilation. At present, it is unclear which strategy is most effective, as few have been directly compared. Our review will use network meta-analysis (NMA) to compare and rank sedation strategies to determine their efficacy and safety for mechanically ventilated patients.

**Methods:**

We will search the following from 1980 to March 2016: Ovid MEDLINE, CINAHL, Embase, PsycINFO, and Web of Science. We will also search the Cochrane Library, gray literature, and the International Clinical Trials Registry Platform. We will use a validated randomized control trial search filter to identify studies evaluating any strategy to optimize sedation in mechanically ventilated adult patients. Authors will independently extract data from eligible studies in duplicate and complete the Cochrane Risk of Bias tool. Our outcomes of interest include duration of mechanical ventilation, time to first extubation, ICU and hospital length of stay, re-intubation, tracheostomy, mortality, total sedative and opioid exposure, health-related quality of life, and adverse events. To inform our NMA, we will first conduct conventional pair-wise meta-analyses using random-effects models. Where appropriate, we will perform Bayesian NMA using WinBUGS software.

**Discussion:**

There are multiple strategies to optimize sedation for mechanically ventilated patients. Current ICU guidelines recommend protocolized sedation or daily sedation interruption. Our systematic review incorporating NMA will provide a unified analysis of all sedation strategies to determine the relative efficacy and safety of interventions that may not have been compared directly. We will provide knowledge users, decision makers, and professional societies with ranking of multiple sedation strategies to inform future sedation guidelines.

**Systematic review registration:**

PROSPERO CRD42016037480

**Electronic supplementary material:**

The online version of this article (doi:10.1186/s13643-016-0338-x) contains supplementary material, which is available to authorized users.

## Background

Sedatives and analgesics are administered in 60–90 % of critically ill patients to manage agitation, reduce pain, and facilitate mechanical ventilation [1–4]. Despite their widespread use, accumulating data indicate these drugs may prolong mechanical ventilation [1, 5–14] and increase delirium [15, 16], long-term cognitive impairment [8, 15], and mortality [10–13]. In this analysis, we will provide a ranking of multiple strategies used to optimize sedation in mechanically ventilated patients to inform future sedation guidelines.

The provision of optimal sedation in critically ill patients is challenging for a number of reasons, including fluctuating illness acuity, the presence of delirium and other disease-associated brain dysfunction (e.g., encephalopathy), pre-morbid psychiatric disorders, and risk of drug or alcohol withdrawal. In addition, multi-organ dysfunction and polypharmacy can render drug pharmacokinetics and dynamics unpredictable, increasing the risk for adverse events and drug interactions. Given these complexities, an ideal sedative or analgesic for critically ill patients would include minimal drug accumulation, ease of titration, tolerable adverse effects, and a clean drug interaction profile (i.e., avoidance of cytochrome P450 metabolism), all at a reasonable cost. At this time however, no sedative or analgesic drug satisfies all these criteria. Despite this, drug administration strategies exist that attempt to mitigate the pharmacokinetic limitations of available drugs; the most common approaches include daily sedation interruption, nurse-directed protocolized sedation, analgesia-based sedation or no sedation, and intermittent dosing versus continuous infusions.

Daily sedation interruption is defined as a short-term suspension, hold, discontinuation, or cessation of intravenous sedative or in some cases, analgesic medication [13]. This approach is used to allow elimination or prevent accumulation of drug, promote patient wakefulness to gauge tolerance for complete cessation of sedation and/or liberation from invasive mechanical ventilation, and where necessary, identify the smallest effective dose of drug to be used. Protocolized sedation involves the titration of sedative and analgesic drugs by intensive care unit (ICU) nurses using a standardized algorithm and sedation assessment scale, most commonly to achieve patient-targeted light sedation [7]. Analgesia-base sedation aims to achieve adequeate analgesia while avoiding the use of sedative drugs [14]. Prioritization of analgesia is important due to the high prevalence of pain and discomfort in the critically ill, which can manifest as agitation. Lastly, continuous intravenous drug infusions [12] permit more stable plasma concentrations, which may facilitate sedation control compared to intermittent dosing; however, continuous infusions can lead to drug accumulation if not titrated closely.

The most recent Society of Critical Care Medicine (SCCM) guidelines [17] recommend protocolized sedation or daily sedation interruption to achieve light sedation in mechanically ventilated ICU patients. These guidelines, however, are based on traditional pair-wise meta-analytic techniques, which only compare two interventions at a time (e.g., daily interruption vs. usual care). Given the growing number of clinical trials and sedation strategies in use, we propose a synthesis of existing data using network meta-analysis (NMA), a novel statistical approach enabling both direct and indirect comparisons in a multi-treatment analytical framework [18–20]. This powerful statistical tool will allow the determination of the relative efficacy and safety of interventions that may or may not have been previously compared head-to-head in randomized controlled trials.

## Methods/design

This review protocol was prepared using the Preferred Reporting Items for Systematic Review and Meta-Analyses Protocol (PRISMA-P) guidelines [21]. We completed the PRISMA-P checklist (Additional file 1). The protocol for this review has been registered using the PROSPERO International Prospective Register of Systematic Reviews (CRD42016037480).

### Data sources and search strategy

Team leaders LB, BH, and LR created a preliminary search strategy (Additional file 2) with an experienced senior information specialist. Prior to execution of the search, a second senior information specialist reviewed the strategy using the Peer Review for Electronic Search Strategies (PRESS) template [22, 23]. The following electronic databases will be searched from1980 to March 2016: Ovid MEDLINE, Ovid MEDLINE In-Process and Other Non-Indexed Citations, CINAHL, Embase Classic+Embase, PsycINFO, and Web of Science. We will not apply a language restriction. We will use a validated randomized controlled trial filter and perform a separate search for published systematic reviews and protocols in the Cochrane Library and PROSPERO. We will form a grey literature search using sources listed in the Canadian Agency for Drugs and Technologies in Health’s (CADTH) Grey Matters [24]. We will search for unpublished and ongoing trials on the International Clinical Trials Registry Platform (http://apps.who.int/trialsearch). We will search the reference lists of relevant articles and systematic reviews for studies not identified through electronic searches. To avoid duplicate study selection, we will compare author names and affiliations, as well as study characteristics, and where uncertainty remains, we will contact corresponding authors for clarification.

### Study eligibility criteria

We will include randomized controlled trials, including those using open-label and quasi-randomized (i.e., quasi-random method of allocation such as alternation) designs. We will include studies that evaluate any sedation strategy in mechanically ventilated adults.

#### Population

Our population of interest is mechanically ventilated, critically ill adults aged 16 years and older and treated in any type of ICU (e.g., burn, cardiac, medical, surgical, trauma, or mixed) or high acuity unit (e.g., high dependency, step-up, step-down units).

#### Intervention

**Fig. 1:**
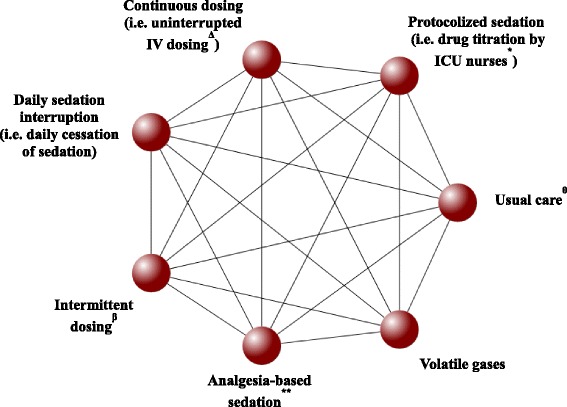
Interventions eligible for network meta-analysis. *Lines* reflect comparisons that may exist between treatments. What comparisons have been studied will be established through identified and included studies. Availability of outcomes can also impact network structure. Clinical experts have guided the network refinement

We will include trials that evaluate any sedation administration strategy in mechanically ventilated adults. Expected interventions include (1) daily sedation interruption (i.e., daily cessation of sedative, and on occasion analgesic drugs), (2) protocolized sedation (i.e., drug titration by ICU nurses using standardized algorithm and assessment tools to targeted patient sedation level), (3) analgesia-based sedation (i.e., opioid managed analgesia, with minimal or no sedative use), (4) intermittent dosing (i.e., bolus dosing, commonl in long acting drugs), (5) continuous dosing (i.e., uninterrupted intravenous dosing, common in short acting drugs), and (6) volatile gases(i.e., volatile anaesthetic agents for sedation). No restrictions will be placed on study selection based on drug class, dose, route or duration of administration. Each intervention will have an individual node in the analytical network (Fig. 1) and additional interventions not specified above will be considered should their studies meet inclusion criteria.

#### Comparators

NMA, an extension of the traditional meta-analytic technique, enables comparisons of multiple interventions based on both direct and indirect evidence [19, 20, 25]. This novel statistical approach will permit the consideration of any trial evaluating any sedation strategies, whether compared to each other or to usual care. Descriptions of usual care will be extracted verbatim from included studies to ensure similarities and differences are appropriately reflected in the network structure used to perform NMA.

#### Outcomes

Our primary outcome is the duration of invasive mechanical ventilation, defined as the time (in days) from intubation to successful extubation, without requirement of re-intubation (or ventilator support in the case of tracheostomy) for a minimum of 24 h. The duration of mechanical ventilation is the primary outcome for most ICU sedation trials.

Secondary outcomes include the following: hospital and ICU length of stay, time to first extubation, tracheostomy, reintubation, mortality, total sedative and opioid exposure (using midazolam and fentanyl equivalents, respectively), use of physical restraints, health-related quality of life, and adverse events (e.g., accidental removal of endotracheal tube or catheter, incidence of delirium, and cardiac events (e.g., ST elevation myocardial infarction (STEMI) or non-STEMI)). As mortality may be defined at various time points, we will extract all reported mortality outcomes and create subgroups where needed for descriptive and analytical purposes.

### Screening and data extraction

Two authors (LB, MG) will independently screen search results against eligibility criteria to identify relevant trials. Prior to screening, we will perform a calibration exercise by piloting the screening tool on a sample of ten studies to ensure consistency in the application of eligibility criteria. References will be organized using the reference management software package EndNote (X7 edition, Thomson Reuters, available at http://endnote.com/). We will examine the full text of any title or abstract selected by either author to determine study inclusion eligibility; in the event of a disagreement, an independent arbiter (LR) will be consulted. Reasons for study exclusion will be documented in the notes field of EndNote. We will report the search strategy and study selection process using a PRISMA flow diagram [26].

We will divide data extraction among pairs of authors (SK/SM, NA/MG, DW/IE). Each member of the author pair will independently extract data using a standardized electronic form that was developed by two authors (BH, MG) in Microsoft Excel version 14.6.2 (Microsoft Corporation, Seattle, WA, USA). The extraction form will be piloted on five studies to ensure its capture of all relevant data (MG, LB). We will extract data related to study design, setting, patient characteristics (e.g., age, gender, severity of illness score, reason for ICU admission), study interventions, co-interventions potentially affecting duration of mechanical ventilation (e.g., spontaneous breathing trials), and stated outcomes of interest.

In the event of missing or unclear data, we will contact study authors directly. Any discrepancies between data extractors will be resolved through discussion with an independent arbiter (LB).

### Risk of bias assessment

Each data extractor will be responsible for assessing the risk of bias of their allocated studies, and a third author (LR) will confirm the final assessment, where necessary. We will use a domain-based evaluation for risk of bias assessment, as recommended by the Cochrane Collaboration [27]. The domains include the following: (1) random sequence generation (i.e., selection bias); (2) allocation concealment (i.e., selection bias); (3) blinding of participants and personnel (i.e., performance bias); (4) blinding of outcomes assessment (i.e., detection bias); (5) incomplete outcome data (i.e., attrition bias); (6) selective reporting; and (7) other bias (e.g., study source of funding). Lack of blinding will not be considered to confer a high risk of bias for objective endpoints such as mortality because the process of blinding is unlikely to influence results.

For each domain, we will assess the risk of bias as “low,” “high,” or “unclear.” Unclear risk will be assigned for a domain if insufficient detail is reported and cannot be obtained from study authors, or if what happened in the study is known, but its contribution to the risk of bias is unknown or unclear. After the assignment of risk of bias, studies will be categorized as follows:

1. Low risk: all domains are considered to be at “low” risk of bias;
2. High risk: one or more domains are considered to be at “high” risk of bias; and
3. Unclear risk: one or more domain(s) have “unclear” risk of bias (and no domain at high risk of bias).

### Approach to evidence synthesis

In the NMA, each intervention will have its own node (Fig. 1). If we encounter combined interventions, the research team will be consulted to determine the best approach for analysis.

Members of the research team will inspect the characteristics of included studies to assess their clinical and methodological homogeneity. We will review the distribution of potential effect modifiers across studies to determine the validity of the assumptions of homogeneity and similarity; [28] these modifiers will likely relate to patient demographics (e.g., age, gender), eligibility criteria (e.g., concomitant medication use), and study design (e.g., blinding, duration of follow-up). For comparisons in the network where a minimum of two studies is available, we will also perform conventional pair-wise meta-analyses using a random-effects model, and derive estimates of between - study heterogeneity of treatment effects (with *I*^2^ > 50 % to be considered indicative of potential importance) [29]. These assessments will permit judgments about whether sufficient homogeneity exists within comparisons in the treatment network. If homogeneity is established, we will progress to performing the planned NMA.

We will perform NMA within a Bayesian framework, assuming a common heterogeneity parameter across all comparisons, and accounting for correlations in multi-arm studies. Analyses will be performed using WinBUGS software [30] (version 1.4.3, MRC Biostatistics Unit, Cambridge, UK) (http://www.mrc-bsu.cam.ac.uk/software/bugs/the-bugs-project-winbugs/) through well-established methods [31, 32]. Continuous and binary outcomes will be expressed in terms of mean differences and odds ratios, respectively, with corresponding 95 % confidence intervals [33–35]. Surface Under the Cumulative Ranking (SUCRA) curve estimates will also be provided [32].

Model adequacy of fit will be evaluated through comparison of the posterior deviance with the number of unconstrained data points (i.e., the total number of intervention arms across studies) [35], and adequacy of fit will be deemed present when these quantities are approximately equal. Both fixed - and random-effects consistency models will be run, and their fit will be examined via comparison of the Deviance Information Criterion (DIC), which penalizes model fit for complexity (note, lower values indicate better models) [36]. A difference of five points or more will be considered indicative of an important difference. We will fit inconsistency models and compare their DIC values with those of consistency models to detect inconsistency. If detected, variations in individual study characteristics will be explored as a potential cause, as will the need for additional statistical considerations such as meta-regression. Model convergence will be assessed using established methods including Gelman-Rubin diagnostics and inspection of Monte Carlo errors [35].

### Subgroup and sensitivity analyses

We will explore subgroup and/or meta-regression analyses to address the impact of covariates on our findings in order to establish their robustness. If sufficient information is identified, we will perform subgroup analyses to determine if our findings are influenced by (1) drug class (e.g., short acting such as propofol vs. long-acting such as benzodiazepines) and (2) type of ICU population (e.g., medical vs. surgical). We will conduct sensitivity analyses excluding studies with high risk of bias and involving alternative geometries of the network. Examples of potential reformulations of the network include consideration of co-interventions known to shorten the duration of mechanical ventilation (e.g., weaning protocols).

### Reporting of review findings

We will adhere to recommendations from the PRISMA-NMA extension statement on NMA for reporting our review findings [37]. We will include recommended graphical approaches such as forest plots, league tables, and rank-o-grams [32]. We will provide a summary of the geometries of the networks to provide insight for future sedation-analgesia trials in mechanically ventilated, critically ill adults.

### Dissemination of findings

We will use the knowledge-to-action framework, which emphasizes the successful implementation of research evidence into practice in two phases: (1) knowledge creation and (2) action. We will seek input from our knowledge users and affiliated organizations for knowledge translation opportunities. We will communicate the findings of this review via a one-page summary tailored to specific audiences (e.g., patients and family members, clinicians, researchers, policy makers), presentations delivered at local, national, and international forums, and publications in peer-reviewed journals. We will hold an end-of-project workshop where key stakeholders will further discuss findings, identify research gaps, and develop a knowledge translation plan to assist with adoption of our findings.

## Discussion

Several sedation administration strategies have been developed to reduce oversedation and duration of mechanical ventilation in critically ill adults. Current critical care guidelines recommend the use of daily sedation interruption or protocolized sedation to achieve targeted light sedation [17]. Although these recommendations are based on a number of trials and systematic reviews, they have yielded mixed results [5, 7, 10, 13, 38–54]. Given the use of additional strategies that have not yet been compared directly (i.e., head-to-head) in clinical trials, we believe the NMA approach has the potential to identify the strategy that most effectively and safely avoids unnecesary days of mechanical ventilation. Despite the strengths of the NMA approach, our study may face certain limitations, such as the reconciliation of different endpoints (e.g., duration of follow-up and mortality measurements) and how to analyze more complex case mixes (e.g., mixed vs. specific ICU patient populations). This data synthesis will nonetheless allow comparisons of all available sedation strategies for mechanically ventilated, critically ill adults, thus providing clinicians, researchers, and professional societies alike with clarity regarding the relative efficacy and safety of available interventions.

### Registration

This systematic review is registered with PROSPERO, an international prospective register of systematic reviews. http://www.crd.york.ac.uk/PROSPERO/display_record.asp?ID=CRD42016037480

## Additional files

Additional file 1: Preferred Reporting Items for Systematic Review and Meta-Analyses Protocol (PRISMA-P). Description: PRISMA-P items and their respective line locations within the manuscript. (DOCX 135 kb) Additional file 2: Preliminary search strategy. Description: Preliminary search strategy, including all queried databases, search parameters, and key words. (DOCX 118 kb)

## Abbreviations

DIC: Deviance Information Criterion
ICU: Intensive care unit
NMA: Network meta-analysis
PRISMA: Preferred Reporting Items for Systematic Reviews and Meta-Analysis
SCCM: Society of Critical Care Medicine
STEMI: ST elevation myocardial infarction
