# Supplementary material for: Comparison of sedation strategies for critically ill patients: a protocol for a systematic review incorporating network meta-analyses
Source: Syst Rev. 2016 Sep 20;5:157. doi: 10.1186/s13643-016-0338-x (PMC5029074; doi:10.1186/s13643-016-0338-x)
Supplement: Additional file 2: — Preliminary search strategy. Description: Preliminary search strategy, including all queried databases, search parameters, and key words. (DOCX 118 kb) [file 13643_2016_338_MOESM2_ESM.docx]

**Additional file 2: Preliminary search strategy**

Database: Ovid MEDLINE(R) In-Process & Other Non-Indexed Citations and Ovid MEDLINE(R) <1946 to Present>, PsycINFO <1806 to March Week 2 2016>, Embase <1980 to 2016 Week 11>

Search Strategy:

--------------------------------------------------------------------------------

1 Intensive Care Units/ (36264)

2 Burn Units/ (1857)

3 Coronary Care Units/ (4019)

4 Respiratory Care Units/ (551)

5 exp Intensive Care Units, Pediatric/ (14486)

6 exp Critical Care/ (44328)

7 ((intensive or critical or acute or subacute or "sub acute") adj3 care).tw. (125980)

8 (ICU or ICUs or NICU or NICUs or PICU or PICUs or SICU or SICUs or CCU or CCUs).tw. (42134)

9 (burn$1 adj3 (unit$1 or centre$1 or center$1)).tw. (3754)

10 ((cardiac or cardiologic or coronary or heart) adj3 (unit$1 or centre$1 or center$1)).tw. (9423)

11 (respiratory adj3 (unit$1 or centre$1 or center$1)).tw. (2942)

12 ((surgical or surger*) adj3 (unit$1 or centre$1 or center$1)).tw. (15105)

13 (("close attention" or "special care") adj (unit$1 or centre$1 or center$1)).tw. (658)

14 Postoperative Care/ (52195)

15 ((postoperati* or post-operati* or postsurg* or post-surg*) adj3 care).tw. (7778)

16 Critical Illness/ (17186)

17 (critical* adj (ill or illness*)).tw. (31379)

18 or/1-17 (263569)

19 exp Respiration, Artificial/ (60072)

20 ((artificial* or mechanical*) adj3 (respirat* or ventilat*)).tw. (42044)

21 (high-frequency adj3 ventilat*).tw. (3077)

22 ((assist* or support* or wean*) adj3 (respirat* or ventilat*)).tw. (16633)

23 ((liquid or fluorocarbon or fluoro-carbon) adj3 ventilat*).tw. (659)

24 ((non-invasive* or noninvasive*) adj3 ventilat*).tw. (4341)

25 controlled ventilation.tw. (1881)

26 (("positive pressure" or "positive end-expiratory pressure") adj3 (respirat* or ventilat*)).tw. (5864)

27 continuous positive airway pressure.tw. (6103)

28 (CPAP or nCPAP).tw. (5956)

29 (airway pressure release adj3 ventilat*).tw. (173)

30 APRV.tw. (110)

31 ((inspiratory or intermittent) adj3 positive pressure breathing).tw. (312)

32 IPPB.tw. (281)

33 Airway Extubation/ (358)

34 exp Intubation, Intratracheal/ (31961)

35 (intubat* or extubat* or detubat*).tw. (47208)

36 or/19-35 (143693)

37 18 and 36 (31439)

38 Conscious Sedation/ (6913)

39 Deep Sedation/ (615)

40 sedat*.tw. (42640)

41 "Hypnotics and Sedatives"/ (22427)

42 or/38-41 (57452)

43 37 and 42 (2754)

44 Midazolam/ (7289)

45 (midazolam or buccolam or dalam or doricum or dormicum or dormonid or fortanest or fulsed or hypnoval or hypnovel or hypnoyvel or ipnovel or midacum or midazo or midazol or midolam or miloz or "Ro 21-3981" or "Ro 213981" or versed).mp. (11855)

46 midazolam.rn. (7289)

47 Propofol/ (11544)

48 (propofol or anepol or aquafol or cryotol or diprivan or disoprivan or disoprofol or fresofol or gobbifol or "ICI-35 868" or "ICI-35868" or ivofol or pofol or propocam or rapinovet or recofol or safol).mp. (16679)

49 propofol.rn. (11544)

50 Lorazepam/ (2587)

51 (Lorazepam* or Apo-Lorazepam or alzapam or anxiedin or anxira or anzepam or aplacasse or aripax or ativan or azurogen or bonatranquan or donix or duralozam or durazolam or efasedan or emotival or idalprem or kalmalin or kendol or larpose or laubeel or lonza or lopam or lorabenz or loram or loranase or loranaze or lorans or lorapam or loravan or lorax or loraz or lorazene or lorazep or lorazin or lorazon or lorenin or loridem or lorivan or lorsedal or lorzem or merlit or mesmerin or nervistop or novhepar or novo-lorazem or Nu-Loraz or orfidal or orifadal or "pro dorm" or punktyl or quait or renaquil or rocosgen or securit or sedatival or sedicepan or sidenar or sinestron or somagerol or stapam or tavor or temesta or titus or tolid or tranquipam or trapax or trapex or upan or WY-4036 or wypax).mp. (4048)

52 lorazepam.rn. (2587)

53 dexmedetomidine/ (1501)

54 (dexmedetomidine or dexdor or dexdomitor or "MPV-1440" or MPV1440 or precedex or primadex).mp. (2474)

55 dexmedetomidine.rn. (1501)

56 Ketamine/ (9359)

57 (ketamine or calipsol or calypsol or "CI-581" or imalgene or kalipsol or katamine or keta-hameln or ketaject or ketalar or ketalin or ketamax or kalipsol or ketalar or ketaminol or ketanest or ketased or ketaset or ketaved or ketavet or ketmin or ketoject or ketolar or narkamon or narketan or "soon-soon" or tekam or velonarcon or vetalar).mp. (14388)

58 ketamine.rn. (9359)

59 Morphine/ (33943)

60 (morphine or aguettant or depodur or dimorf or duramorph or duromorph or epimorph or "l-Morphine" or "M-Eslon" or miro or morfina or morphia or morphin or morphina or morphinum or morphium or moscontin or "MS Contin" or nepenthe or opso or "oramorph SR" or roxanol or "SDZ 202-250" or "SDZ202-250" or sevredol or skenan).mp. (49113)

61 morphine.rn. (33943)

62 Hydromorphone/ (1030)

63 (hydromorphone or biomorphyl or cofalaudid or dihydromorphinone or di-hydromorphinone or dihydromorphone or di-hydromorphone or diladid or dilaudid or dimorphone or dolonovag or dimo or dimorphone or exalgo or hydromorph contin or hydromorphinone or hydromorphone or hydrostat or hymorphan or jurnista or laudaconum or novolaudon or opidol or paliadon or retardkaps or palladon or palladone or semcox or sophidone).mp. (1614)

64 hydromorphone.rn. (1030)

65 exp Fentanyl/ (13408)

66 (fentanyl or abstral or duragesic or durogesic or durotep or fentanest or fentamyl or fentanylum or fentora or IONSYS or lazanda or leptanal or matrifen or mhentanyl or onsolis or pecfent or phentanyl or rapinyl or recuvyra or "R-4263" or sublimase or sublimaze or subsys or tanyl or transfenta).mp. (18005)

67 fentanyl.rn. (11496)

68 (Remifentanil or "GI 87084B" or GI87084B or Ultiva).tw. (3248)

69 remifentanil.rn. (2409)

70 Clonazepam/ (2297)

71 (antelepsin or antilepsin or apetryl or chlonazepam or cloazepam or clonazepam* or clonex or clonopin or iktorivil or kenoket or klonopin or landsen or lktorivil or lonazep or melzap or paxam or rivotril or Solfidin or "BRN 0759557" or "EINECS 216-596-2" or "HSDB 3265" or "NSC 179913" or "Ro 4-8180" or "Ro 5-4023" or "Ro 54023" or "UNII-5PE9FDE8GB").tw. (3052)

72 clonazepam.rn. (2297)

73 Analgesics, Opioid/ (28925)

74 ((narcotic* or opioid*) adj3 analgesi*).tw. (8484)

75 or/44-74 (125657)

76 37 and 75 (1823)

77 43 or 76 (3487)

78 (controlled clinical trial or randomized controlled trial or pragmatic clinical trial).pt. (464810)

79 clinical trials as topic.sh. (170168)

80 (randomi#ed or randomly or RCT$1 or placebo*).tw. (642079)

81 ((singl* or doubl* or trebl* or tripl*) adj (mask* or blind* or dumm*)).tw. (131887)

82 trial.ti. (131095)

83 or/78-82 (963949)

84 77 and 83 (761)

85 exp Animals/ not (exp Animals/ and Humans/) (3967499)

86 84 not 85 (749)

87 (comment or editorial or interview or news).pt. (1034000)

88 (letter not (letter and randomized controlled trial)).pt. (853101)

89 86 not (87 or 88) (739)
